# Macronutrient Balance and Dietary Glycemic Index in Pregnancy Predict Neonatal Body Composition

**DOI:** 10.3390/nu8050270

**Published:** 2016-05-06

**Authors:** Nathalie V. Kizirian, Tania P. Markovic, Roslyn Muirhead, Shannon Brodie, Sarah P. Garnett, Jimmy C. Y. Louie, Peter Petocz, Glynis P. Ross, Jennie C. Brand-Miller

**Affiliations:** 1 Charles Perkins Centre, The University of Sydney, Sydney 2006, NSW, Australia; Nathalie.kizirian@sydney.edu.au (N.V.K.); roslyn.muirhead@sydney.edu.au (R.M.); shannon.brodie@sydney.edu.au (S.B.); jimmy.louie@sydney.edu.au (J.C.Y.L.); 2 School of Life and Environmental Sciences, The University of Sydney, Sydney 2006, NSW, Australia; 3 Boden Institute of Obesity, Nutrition, Exercise & Eating Disorders, The University of Sydney, Sydney 2006, NSW, Australia; tania.markovic@sydney.com; 4 Department of Endocrinology, Royal Prince Alfred Hospital, Camperdown 2050, NSW, Australia; gpross@bigpond.net.au; 5 Institute of Endocrinology and Diabetes, The Children’s Hospital at Westmead, Sydney 2145, NSW, Australia; sarah.garnett@health.nsw.gov.au; 6 The Children’s Hospital at Westmead Clinical School, University of Sydney, Sydney 2006, NSW, Australia; 7 Department of Statistics, Macquarie University, Sydney 2109, NSW, Australia; peter.petocz@mq.edu.au; 8 Sydney Medical School, University of Sydney, Sydney 2006, NSW, Australia

**Keywords:** maternal, pregnancy, dietary intake, nutrition, macronutrient, gestational diabetes mellitus, Pea Pod, neonates, body composition

## Abstract

The influence of maternal macronutrient balance and dietary glycemic index (GI) on neonatal body composition has received little study. We hypothesized that the overall quantity and quality of macronutrients, particularly carbohydrate, in the maternal diet could have trimester-specific effects on neonatal growth and body composition in women at risk of gestational diabetes. Maternal diet was assessed using 3-day food records in mid (*n* = 96) and late (*n* = 88) pregnancy as part of the GI Baby 3 study. Neonatal body composition was assessed by air-displacement plethysmography within 48 h of birth, adjusted for length, and expressed as fat mass index (FMI) and fat-free mass index (FFMI). In mid pregnancy, higher maternal intake of carbohydrate energy was negatively correlated with infant FFMI (*p* = 0.037). In late pregnancy, higher dietary GI was associated with lower FFMI (*p* = 0.010) and higher carbohydrate energy predicted lower FMI (*p* = 0.034). Higher fat intake (%E) and saturated fat, but not protein, also predicted neonatal body composition (higher FFMI in mid pregnancy and higher FMI in late pregnancy). Depending on pregnancy stage, a high carbohydrate-low fat diet, particularly from high glycemic sources, may reduce neonatal indices of both lean mass and adiposity.

## 1. Introduction

Prenatal nutrition plays a critical role in defining offspring health [1,2]. Optimal fetal growth depends on adequate maternal nutrient supply at each specific stage of fetal development. Studies from the Dutch famine showed that exposure to famine in early but not late pregnancy was associated with increased obesity risk and markers of the metabolic syndrome in adult life, suggesting that prenatal undernutrition has long-term effects that depend on its timing [3,4]. It is conceivable that dietary extremes at the time of conception (but not later) have more effect on tissue differentiation and proliferation while diet in later pregnancy may influence fetal adiposity [5,6]. Birth weight has long been recognized as a determinant for disease in later life [7,8]. However, size at birth is influenced by multiple factors, including genetic inheritance, maternal constraints [9], maternal metabolism [10,11] and smoking [12], making birth weight a poor surrogate for disease risk in adult life. Recent evidence indicates that neonatal body composition is a more sensitive marker of intrauterine environment than birth weight and a better predictor of long-term health outcomes [13,14].

Despite the strong link between maternal diet and offspring health, the effect of macronutrient distribution on neonatal growth and body composition has not been explored. One of the main challenges in determining the optimal maternal diet lies in the fact that diets are a complex combination of foods and focusing on single nutrients fails to account for the interactions between nutrients [15]. Additionally, much of the evidence highlighting the importance of prenatal nutrition in influencing fetal growth comes from interventional studies of dietary supplementation in undernourished groups [2]. Compelling evidence in animal models suggests that, rather than macronutrients acting individually, their interactive effects (balance) influence long-term health [16,17]. In humans, maternal dietary balance has been shown to dictate fetal adiposity and fat distribution, assessed by ultrasound at 36 weeks of gestation [18]. Furthermore, as maternal diet can induce epigenetic changes [5] and affect long-term body composition [19], understanding the interaction between maternal nutrition balance and offspring body composition could provide insight on long-term disease susceptibility.

The aim of this study was therefore to explore the associations between maternal macronutrient balance, carbohydrate quality (glycemic index, GI) and neonatal body composition, in women at risk of gestational diabetes mellitus (GDM). Maternal dietary intake was assessed at two separate time points (mid and late pregnancy). We hypothesized that nutrient supply would have different effects on neonatal body composition, depending on the timing of pregnancy. We used data derived from the GI Baby 3 study, a two-arm parallel randomized controlled trial assessing the effects of a low glycemic index (GI) compared to a high fiber diet in pregnancy on perinatal outcomes [20].

## 2. Materials and Methods

### 2.1. Sample

This ecologic study is a secondary analysis of the GI Baby 3 study, a two-arm randomized controlled trial, assessing the effects of a low GI diet compared to a conventional high fibre diet during pregnancy on perinatal outcomes (*n* = 139) [20]. Women with singleton pregnancy who were at risk of developing GDM were recruited at 14–20 weeks gestation from the antenatal clinic at the Royal Prince Alfred Hospital, Sydney, Australia. Women were eligible if they had at least one of the following risk factors for GDM: pre-pregnancy BMI ≥30 kg/m^2^, age ≥35 years, polycystic ovary syndrome, previous history of GDM, previous history of newborn >4000 g, family history of type 2 diabetes (first degree relatives), belonging to an ethnic group with high prevalence of GDM (Aboriginal, Torres Strait Islander, Polynesian, Middle Eastern, Indian, Asian). The GI Baby 3 study focused on women clinically identified as being at risk of developing GDM as this group is more likely to show elevated blood glucose concentrations during pregnancy. Exclusion criteria were special dietary requirements (gluten-intolerant, celiac disease), pre-existing diabetes, unable to understand English or to comply with scheduled visits during pregnancy (5 visits). A total of 139 women joined the study and 125 completed the study (34–36 weeks of gestation). The GI Baby 3 study was conducted in accordance with the ethical standards of the Human Research Ethics Committee of the Sydney South West Area Health Service (Royal Prince Alfred Hospital zone; reference no. HREC/10/RPAH/453). All participants gave written informed consent.

### 2.2. Data Collection

Maternal demographics were collected at the first visit (week 14–20 of gestation). Women underwent an oral glucose tolerance test (OGTT) at study entry (<20 weeks gestation). In women free from GDM, a second OGTT was undertaken at 26–28 weeks of gestation. GDM diagnosis was based on the 1998 Australasian Diabetes in Pregnancy Society criteria [21]. Maternal insulin sensitivity index (ISI) was calculated according to the Matsuda and deFronzo formula [22]. Gestational weight gain (GWG) was computed as the difference between measured weight at 34–36 weeks gestation and self-reported pre-pregnancy weight and categorized according to the 2009 Institute of Medicine (IOM) recommendations [23]. Information on perinatal outcomes was obtained from medical records. Maternal dietary intake was assessed using 3-day food records (two week-days and one weekend day) at week 14–20 of gestation, referred to as mid pregnancy (diet prior to the dietary intervention), and at week 34–36 of gestation, referred as late pregnancy (diet post dietary intervention). Written and verbal instructions on how to complete the 3-day food records was provided by the research dietitian. Participants were asked to weigh or measure everything they consumed over the three days using kitchen scales or household implements (e.g., metric measuring cups or spoons), and to note product brand names, cooking methods, and provide recipes where relevant. Completed food diaries were reviewed by the dietitian in consultation with the subjects, and uncertainties with regards to portion size were clarified using visual aids such as measuring cups. All women were advised to increase their intake of fibre-rich foods including wholegrains, vegetables and fruit. Women in the low GI group were specifically encouraged to consume the low GI versions of these foods. Dietary data was analyzed using the computer program “Food Works Professional” (FoodWorks 7 Professional; Xyris Software, Brisbane, QLD, Australia) based on the Australian food composition database AUSTNUT2007. Dietary GI values were assigned to carbohydrate food items using published sources [24] and the University of Sydney GI Research Service database. Validity of the 3-day food records was assessed using the Goldberg cut-off [25] where energy intake (EI) was obtained from the food records and the basal metabolic rate (BMR) was estimated using the Schofield equation [26]. Physical activity levels were estimated as 1.6 in mid and late pregnancy. Applying this method, adequate dietary data was reported in 85 (89%, EI:BMR 1.04–2.47) and 74 (84%, EI:BMR 1.04–2.47) women in mid and late pregnancy, respectively. Removing the under/over-reporters had no material effect on the findings so they were retained in the final analysis.

Neonatal anthropometry was obtained from electronic medical records. Birth length was measured by experienced midwives in the delivery ward, at the time of birth as part of routine clinical care, using a Seca measuring mat for infants. Crown to heel length was re-measured within the first 48 h of birth by an endocrinologist (TPM) and one of the two research dietitians (RM, SB) for Pea Pod measurement, using a neonatometer. Infant weight-for-age *z*-score was calculated using gender-specific reference database from the World Health Organization (WHO Anthro for personal computers software, version 3.2.2, Geneva, Switzerland) [27]. Ponderal index was calculated as birth weight (g) divided by length (cm)^3^ × 100. Neonatal body composition was assessed within 48 h after birth, using an air-displacement plethysmograph device (PEA POD, COSMED, Concord, CA, USA) [28]. Infant hair was smoothed down with water to minimize air behaving isothermally.

### 2.3. Statistics

Primary outcome was neonatal body composition, expressed as fat mass index (FMI; FM (kg)/length (m)^2^) and fat-free mass index (FFMI; FFM (kg)/length (m)^2^). By adjusting for neonatal length, FM and FFMI have been demonstrated to be the best proxy for infant body composition [29,30]. Normally distributed data are presented as means ± standard deviation (SD) and number (*n*) and percentage (%) for frequency variables. Non-normally distributed data are presented as median, 25th and 75th percentiles. Multiple linear regression analyses were used to assess the effect of maternal macronutrient intake in mid and late pregnancy on infant body composition at birth, expressed as FMI and FFMI. The analyses were adjusted for maternal pre-pregnancy BMI, GDM, gender and gestational age (significance set at *p* < 0.05). Adjusted *R*^2^, beta-value and 95% confidence intervals (CI) are reported. Statistical analyses were performed using SPSS Version 21 (IBM Australia, St Leonards, NSW, Australia).

The geometric framework [31] is a state-space nutritional modeling method used to explore interactive effects of maternal macronutrient intake (expressed as percentage of total energy, %E) on infant body composition. This approach allowed visualization of the complex relation between one outcome variable (FMI or FFMI) and two macronutrients in the maternal diet, plotted on the x and y axis. Infant body composition is represented by isolines, rising from dark blue (representing the lowest values) to dark red (representing the highest values). The graphics were drawn using the R software (R 3.0, the R Project Statistical Computing). Bland Altman was used to assess the agreement between birth length and re-measured length for Pea Pod measurement.

## 3. Results

**Figure 1:**
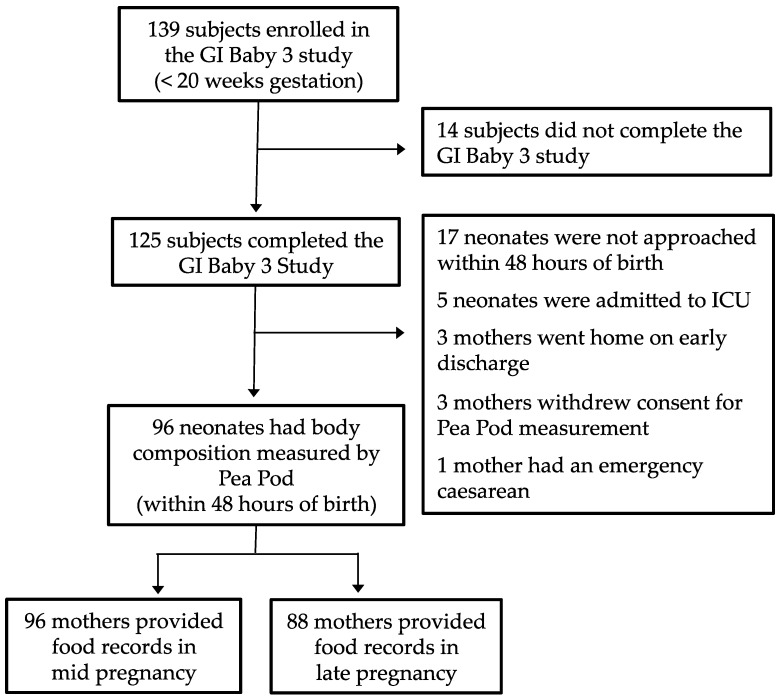
Flow of participants through the glycemic index (GI) Baby 3 Study.

Of the 139 women enrolled in the GI Baby 3 study, 125 completed the protocol. Of these, 96 neonates (77%) had body composition assessment within 48 h after birth. The measurement error between birth length and re-measured length was 0.78 mm, limits of agreement 48.7 to 51.7 mm and intra-class correlation was 0.894, 95% CI 0.841 to 0.929, *p* < 0.001. Completed food records were available for 96 and 88 mothers in mid and late pregnancy, respectively (Figure 1). Maternal and neonatal characteristics are presented in Table 1.

Dietary data in mid and late pregnancy for women with neonatal body composition assessment are presented in Table 2.

### Maternal Macronutrient Balance and Neonatal Body Composition

Multiple linear regression analyses of associations between maternal diet in mid pregnancy and neonatal body composition are summarized in Table 3.

In mid pregnancy, only carbohydrate, total fat and saturated fat displayed significant relationships. Carbohydrate energy (%E) was negatively correlated with offspring FFMI, explaining ~10% (*p* = 0.037) of the variation. Conversely, total fat (%E) and saturated fat (%E) in mid pregnancy were both positively correlated with offspring FFMI, explaining ~12% (*p* = 0.012) and ~13% (*p* = 0.010) of the variation, respectively.

**Figure 2:**
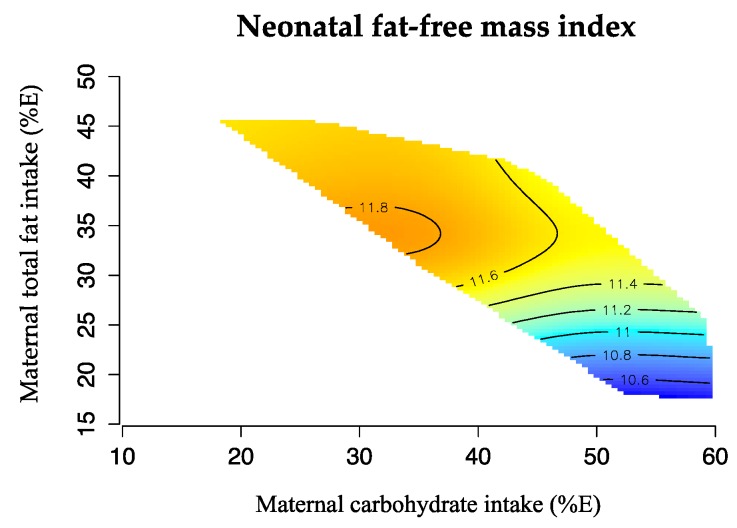
Effects of maternal carbohydrate (%E) and total fat (%E) intake in mid pregnancy and offspring FFMI. The isolines for the FFMI rise in elevation from dark blue to dark red. Neonatal FFMI was greatest at low proportions of dietary carbohydrate (<55%E) and moderate fat (>30%E) intakes (*n* = 96).

Figure 2 illustrates these associations using ‘response surfaces’. Neonatal FFMI was greatest when maternal dietary carbohydrate was <55%E and fat intakes were >30%E.

In contrast to FFMI, there were no significant associations between neonatal FMI and maternal dietary intake in mid pregnancy. Similarly, there were no significant associations with protein intake (%E), P:C ratio or dietary fiber in mid pregnancy with neonatal FMI or FFMI.

Table 4 summarizes the findings in late pregnancy after women had received nutrition education for at least 16–20 weeks. This included dietary GI advice (either low or moderate GI depending on treatment group) and instruction to follow a healthy diet.

**Figure 3:**
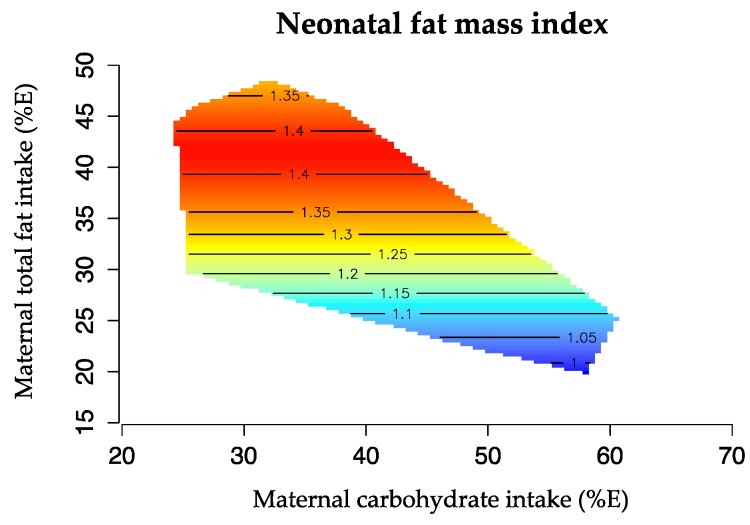
Effects of maternal carbohydrate (%E) and total fat intake (%E) in late pregnancy and neonatal FMI. The isolines for the FMI rise in elevation from dark blue to dark red. Offspring FMI was greatest at high maternal intake of total fat (>40%E) and moderate intake of carbohydrate (<45%E) (*n* = 88).

Maternal macronutrient balance in late pregnancy was associated with differences in both neonatal FMI and FFMI. Higher carbohydrate intake (%E) was associated with lower FMI, explaining ~12% of the variance (*p* = 0.034), while higher intakes of total fat (%E) and saturated fat (%E) predicted higher FMI, explaining ~12% of the variation (*p* = 0.032 and *p* = 0.033 respectively). Thus, offspring FMI was greatest at relatively low intake of carbohydrate (<45%E) and relatively high intake of fat (>40%E, Figure 3).

**Figure 4:**
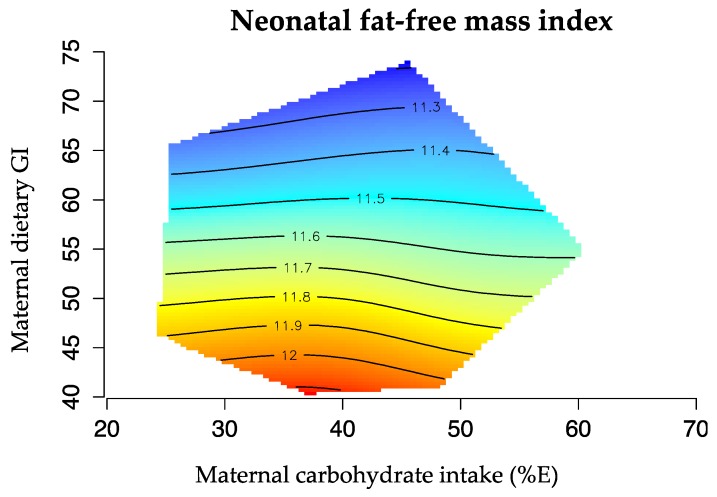
Effects of maternal carbohydrate intake (%E) and dietary GI in late pregnancy and offspring FFMI. The isolines for the FMI rise in elevation from dark blue to dark red. FFMI was lowest at high proportions of carbohydrate intake (>50%E) derived from high GI sources (>50 GI) (*n* = 88).

In addition, in late pregnancy, high proportions of carbohydrate (>50%E), particularly from high GI sources, predicted lower neonatal FFMI as illustrated in Figure 4. Dietary GI alone explained 11% of the variation in FFMI (*p* = 0.010).

There were no significant associations between maternal protein intake (%E), P:C ratio or dietary fiber in late pregnancy with either FMI or FFMI.

## 4. Discussion

In this study, we explored a large database containing data on maternal dietary intake and infant body composition assessed by air displacement plethysmography within 48 h of birth. We hypothesised that the overall quantity and quality of macronutrients in the maternal diet, particularly of carbohydrate, could have trimester-specific effects on neonatal growth and body composition. Our findings suggest that diet composition *per se* may indeed influence fetal tissue growth and that the effects may differ from early to late pregnancy. In mid pregnancy, we found that a higher carbohydrate diet containing less total and/or saturated fat as a proportion of total dietary energy was associated with lower indices of neonatal fat-free mass, while. Thus, depending on pregnancy stage, a high carbohydrate-low fat diet, particularly from high glycemic sources, may reduce neonatal indices of both lean mass and adiposity.

To explain these findings, we speculate that the circulating fuels in the mother’s bloodstream (glucose, free fatty acids, amino acids and possibly ketones) will influence the fetal tissue responses and therefore tissue growth. Higher blood glucose levels in the mother, will stimulate hyperinsulinaemia in the fetus and thereby stimulate the growth of lean or adipose tissue, depending on the stage of pregnancy [32]. In human infants, the majority of adipose tissue is laid down in the 3rd trimester. It is likely that high levels of free fatty acids (the building blocks of triglycerides) in the mother’s blood stream will encourage adipose tissue growth but not lean tissue [33]. Maternal intake of saturated fatty acids, but not monounsaturated or polyunsaturated fatty acids, has been associated with greater fetal adiposity, but the mechanisms remain unclear [18].

Our results suggest that the quantity and quality of maternal macronutrient intake during pregnancy may be more important contributors to fetal growth and body composition than is currently recognised. Although cross-sectional studies cannot prove cause-and-effect, these findings in a group of women at risk of GDM have important implications. Low fat mass at birth is directly related to higher morbidity in the first weeks of life [34] and may also be linked to higher risk of cardiovascular disease in later life [35]. At the other extreme, excessive body fat in the neonate (macrosomia) is associated with adverse pregnancy outcomes and increased susceptibility to obesity and metabolic disease in adult life [36].

Although there are few previous studies of similar nature, their findings are consistent in part with ours. Blumfield *et al.* reported greater fetal adiposity at the midthigh associated with low carbohydrate (<40%E) intake and high fat (>40%E) in a large group of Australian mother-infant pairs who had ultrasound measurements of abdominal and thigh circumference at 36 weeks gestation [18]. In a secondary analysis of the ROLO study (randomised control trial of low glycaemic index diet *versus* no dietary intervention to prevent recurrence of fetal macrosomia), neonatal abdominal adiposity was positively associated with maternal saturated fat intake [37]. In a British cohort, lower maternal carbohydrate intake during pregnancy was associated with greater child adiposity at 9 years of age [19]. Finally, animal models also indicate that a high-fat diet during gestation will produce offspring that develop increased adiposity or reduced lean mass, independent of maternal obesity [38].

In the present study, we found trimester specific effects of dietary carbohydrate. In mid pregnancy, carbohydrate energy appeared to influence only FFMI, while in late pregnancy it predicted only FMI. Furthermore, in late pregnancy but not mid pregnancy, higher dietary GI was associated with lower FFMI. Indeed, the GI alone explained a similar amount of the variation (11%) in lean mass as carbohydrate. This implies that higher intake of carbohydrate foods with a high GI, such as bread, rice and potatoes, might act to reduce fetal lean mass accumulation. Because lean mass is a primary determinant of basal metabolic rate, the combined effects of a high carbohydrate, high GI diet throughout pregnancy may increase offspring predisposition to weight gain in later life.

Unlike previous studies, we did not find a relationship between maternal protein intake and the growth and body composition of her fetus. This may have resulted from the relatively high (~20%E) and narrow range of protein intake in our participants. Furthermore, unlike previous studies, we did not find a relationship between the maternal protein to carbohydrate (P:C) ratio and body composition. Blumfield *et al.* reported that a higher ratio (more protein and less carbohydrate) was associated with lower fetal abdominal fat mass assessed by fetal ultrasound measurements at ~36 weeks [18]. Our sample of mothers, however, had a very high ratio (P:C ratio = 1:2) that may have reduced the ability to detect a significant relationship. It should also be acknowledged that the effects of maternal diet on fetal body composition may differ between pregnancies from general population (such as in the WATCH cohort [18]) and pregnancies at high risk of developing GDM.

Total energy intake during pregnancy also plays a role in influencing fetal growth and birth weight [2]. However, we did not find a significant association between maternal energy intake and offspring body composition. Energy intake in our sample was lower than the results reported in a recent review on energy and macronutrient intakes during pregnancy [39]. Indeed, mean energy intakes during pregnancy pooled from 7 different studies conducted in Australia was ~9260 ± 1100 kJ while our sample reported a mean energy intake of 8570 ± 1440 kJ. Furthermore, our mothers sample reported a decrease in energy intake with advancing pregnancy [39]. These observations may be related to the fact that all of our participants had risk factors for the development of GDM (including high pre-pregnancy BMI, family history or previous macrosomic infant) and were therefore cautioned to avoid excessive weight gain.

The relative strengths and weaknesses of our study should be noted. We benefited from a large dataset of women whose dietary intake had been recorded using 3-day records at specific stages of pregnancy. Trained dietitians checked and analysed the data. Neonatal body composition was assessed within 48 h of birth by air-displacement plethysmography, a method validated against the 4-compartments model [28] and deuterium dilution [40]. Primary outcomes were expressed as FMI and FFMI, which adjust tissue masses for body length while keeping FM and FFM outcomes separate. These indices are recognized as the most appropriate approach to evaluating pediatric body composition [29,30]. Using %FM as an index of fatness has been suggested to be misleading as it ignores between-subject variation in FFM. For example, infants will differ in %FM if they have identical FFM but different FM or if they have identical FM but different FFM [29]. Finally, we used the geometric framework to capture multidimensional aspects of nutrition. This novel approach reveals interactions that may not be evident when macronutrients are examined in two-dimensional space.

The limitations of our study must also be acknowledged. Under-reporting is an important concern, especially among overweight and obese women. Mean energy intake was 8.8 and 8.3 MJ in mid and late pregnancy respectively, which is lower than that reported by others [41,42]. Dietary data collected over 3-days for the purposes of a study may not be a reliable representation of actual food intake over the whole trimester. Moreover, it is possible that variation between individuals is actually variation from day-to-day (intra-individual variation) and variability may be higher for some nutrients *versus* others. Our participants were at risk of developing GDM and received intensive nutrition education to improve the quality of their diet which means our findings may not be generalized to the normal population of pregnant women. The majority of the participants in the GI Baby 3 study had a tertiary education and their food intake, particularly the relatively high proportion of protein, may not be representative.

FMI and FFMI are suggested to be the best proxy for body composition assessment, providing reliability and accuracy in body length measurement. However, neonatal crown to heel length is not easy to measure precisely and a lack of accuracy may cause distortion to the final result of the equation. Findings relying on these indices should be interpreted with caution. Maternal metabolic state will also influence neonatal body composition and potentially override the effects of maternal diet. Indeed, insulin resistance in the first half of pregnancy, and glycemia in the second half of pregnancy have been shown to be highly predictive of newborn adiposity [43]. Finally, our statistical analysis is prone to type 1 error because multiple relationships have been tested. Causality cannot be inferred from this study due to its observational nature and the possibility of residual confounding.

## 5. Conclusions

A better understanding of the effects of maternal nutrition in early life and its influence on short- and long-term health should be a priority area of study in nutrition research. This analysis raises questions and hypotheses for further study. In women at high risk of diabetes, neonatal body composition may be partially driven by maternal dietary balance. Depending on pregnancy stage, a high carbohydrate-low fat diet, particularly from high glycemic sources, may reduce neonatal indices of both adiposity and lean mass. At one extreme, a very high carbohydrate diet may compromise the ability to optimise fetal body composition with adequate lean tissue and adipose tissue stores for life outside the womb. At the other extreme, a very low carbohydrate diet with higher fat/saturated fat content may predispose the infant to macrosomia with corresponding adverse effects on pregnancy outcomes and later risk of obesity.

## Figures and Tables

**Table 1:** Maternal and neonatal characteristics (*n* = 125).

Characteristic	Value
Age (year)	34.8 ± 4.3
Pre-pregnancy BMI (kg/m^2^)	25.2 ± 5.3
BMI ≥ 25 kg/m^2^	45 (36.0)
Ethnicity	
Caucasian	72 (57.6)
Asian	33 (26.4)
Others	20 (16.0)
Tertiary education	95 (76.0)
Nulliparous	59 (47.2)
GWG (kg)	10.9 ± 5.5
IOM weight gain range	
Below	44 (35.8)
Within	51 (41.5)
Above	28 (22.8)
Delivery	
Vaginal delivery	89 (71.2)
Elective caesarean	18 (14.4)
Emergency caesarean	18 (14.4)
GDM	38 (30.4)
GDM diagnosed	
<20 weeks gestation	20 (16)
26–28 weeks gestation	18 (14.4)
Insulin use	23 (18.4)
HbA1c (%) ^1^	4.9 (0.3)
ISI ^2^	9.9 (4.8)
Gestational age ^3^	39.6 (38.7, 40.4)
Male	39.2 (35, 42)
Female	39.8 (35.6, 41.5)
Sex (male)	60 (48)
Birth weight (kg)	3.4 ± 0.4
Male	3.4 ± 0.5
Female	3.5 ± 0.4
Birth length (cm) ^3^	50.0 (49, 51)
Male	50 (43.5, 55.0)
Female	50.5 (45.0, 57.0)
Weight-for-age *z*-score	0.2 ± 0.9
Male	0.0 ± 1.0
Female	0.4 ± 0.8
Ponderal Index (kg/m^3^)	2.7 ± 0.2
Male	2.7 ± 0.2
Female	2.7 ± 0.2
%FM ^4^	10.1 ± 3.8
Male	8.8 ± 3.3
Female	11.2 ± 3.9
FMI ^3,4^	1.3 (0.9, 1.6)
Male	1.2 ± 0.5
Female	1.5 ± 0.6
FFMI ^4^	11.6 ± 0.9
Male	11.8 ± 1.1
Female	11.5 ± 0.8

Mean ± SD (all such values); *n* (%) (all such values). ^1^ Data available in 123 subjects; ^2^ data available in 115 subjects; ^3^ median (25th, 75th percentiles) (all such values); ^4^ data available in 96 infants, 48 male and 52 female. GWG, gestational weight gain; IOM, Institute of Medicine; GDM, gestational diabetes mellitus.

**Table 2:** Maternal daily dietary intake in mid and late pregnancy of GI Baby 3 participants whose infants had assessment of body composition within 48 h of birth.

Heading	Mid Pregnancy	Late Pregnancy
*n*	96	88
Energy (MJ)	8.8 ± 1.9	8.3 ± 1.6
Protein (g)	100.0 ± 23	97.0 ± 28.2
Total fat (g)	80.6 ± 22.8	77.5 ± 24.3
Total carbohydrates (g)	233.6 ± 64.3	210.3 ± 47.0
Sugars (g)	95.4 ± 36	89.0 ± 27.5
Starch (g)	136.6 ± 48	120.0 ± 31.3
Fiber (g)	25.8 ± 8.3	27.0 ± 8.6
P:C ratio	0.5 ± 0.2	0.5 ± 0.2
GI	57 ± 5	54 ± 6
GL	125 ± 41	106 ± 30
Protein (%E)	19.5 ± 4.1	20.0 ± 4.1
Total fat (%E)	33.6 ± 5.7	34.2 ± 6.0
Saturated fat (%E)	12.7 ± 3.0	12.3 ± 3.0
Carbohydrates (%E)	43.5 ± 6.5	42.1 ± 6.4

Mean ± SD (all such values). P:C ratio, protein-to-carbohydrate ratio; GI, glycemic index; GL, glycemic load; %E, percentage of total energy.

**Table 3:** Maternal daily dietary intake in mid pregnancy and offspring fat-free mass index (FFMI) and fat mass index (FMI) (*n* = 96).

	*R*^2^	Beta	95% CI	*p*
**FFMI**				
Energy (MJ)	0.063	0.035	−0.071, −0.141	0.517
Protein (%E)	0.059	−0.003	−0.050, 0.043	0.883
Total fat (%E)	0.123	0.041	0.009, 0.073	**0.012**
Saturated fat (%E)	0.126	0.079	0.019, 0.139	**0.010**
Carbohydrates (%E)	0.104	−0.030	−0.057, −0.002	**0.037**
Fiber (%E)	0.069	−0.144	−0.443, 0.154	0.339
P:C ratio	0.067	0.471	−0.569, 1.512	0.371
GI	0.064	−0.012	−0.046, 0.023	0.503
GL	0.070	−0.002	−0.007, 0.002	0.312
**FMI**				
Energy (MJ)	0.073	0.046	−0.017, 0.108	0.151
Protein (%E)	0.075	−0.021	−0.048, 0.007	0.134
Total fat (%E)	0.076	0.015	−0.004, 0.034	0.131
Saturated fat (%E)	0.067	0.022	−0.015, 0.059	0.235
Carbohydrates (%E)	0.055	−0.004	−0.021, 0.013	0.615
Fiber (%E)	0.059	0.074	−0.104, 0.252	0.413
P:C ratio	0.056	−0.205	−0.826, 0.416	0.513
GI	0.052	0.002	−0.019, 0.022	0.875
GL	0.054	0.001	−0.002, 0.003	0.643

Multiple linear regression, adjusted for maternal pre-pregnancy BMI, GDM, gender and gestational age. %E, percentage of total energy; P:C ratio, protein-to-carbohydrate ratio; GI, glycemic index; GL, glycemic load.

**Table 4:** Maternal daily dietary intake in late pregnancy and offspring FFMI and FMI (*n* = 88).

	*R*^2^	Beta	95% CI	*p*
**FFMI**				
Energy (MJ)	0.037	0.020	−0.106, 0.147	0.750
Protein (%E)	0.038	−0.011	−0.063, 0.041	0.681
Total fat (%E)	0.045	0.015	−0.018, 0.049	0.369
Saturated fat (%E)	0.040	0.020	−0.045, 0.084	0.544
Carbohydrates (%E)	0.047	−0.017	−0.051, 0.017	0.330
Fiber (%E)	0.040	0.072	−0.175, 0.319	0.564
P:C ratio	0.036	0.126	−1.098, 1.349	0.839
GI	0.110	−0.040	−0.071, −0.010	**0.010**
GL	0.064	−0.006	−0.013, 0.001	0.118
**FMI**				
Energy (MJ)	0.079	0.026	−0.049, 0.100	0.495
Protein (%E)	0.073	−0.003	−0.034, 0.027	0.835
Total fat (%E)	0.124	0.021	0.002, 0.040	**0.032**
Saturated fat (%E)	0.124	0.040	0.003, 0.077	**0.033**
Carbohydrates (%E)	0.123	−0.021	−0.042, −0.002	**0.034**
Fiber (%E)	0.084	0.070	−0.074, 0.214	0.336
P:C ratio	0.090	0.437	−0.272, 1.147	0.224
GI	0.073	0.001	−0.018, 0.020	0.927
GL	0.084	−0.002	−0.006, 0.002	0.322

Multiple linear regression, adjusted for maternal pre-pregnancy BMI, GDM, gender and gestational age. %E, percentage of total energy. P:C ratio, protein-to-carbohydrate ratio. GI, glycemic index. GL, glycemic load.
